# Breast Cancer (BC) Is a Window of Opportunity for Smoking Cessation: Results of a Retrospective Analysis of 1234 BC Survivors in Follow-Up Consultation

**DOI:** 10.3390/cancers13102423

**Published:** 2021-05-17

**Authors:** Marion Nicolas, Beatriz Grandal, Emma Dubost, Amyn Kassara, Julien Guerin, Aullene Toussaint, Enora Laas, Jean-Guillaume Feron, Virginie Fourchotte, Fabrice Lecuru, Noemie Girard, Florence Coussy, Beatrice Lavielle, Irene Kriegel, Youlia Kirova, Jean-Yves Pierga, Fabien Reyal, Anne-Sophie Hamy

**Affiliations:** 1 Faculty of Medicine, Sorbonne Université, 75006 Paris, France; marion.nicolas@aphp.fr; 2 Department of Breast and Gynecological Surgery, Institut Curie, Université Paris, 75005 Paris, France; beatriz.grandalrejo@curie.fr (B.G.); emma.dubost@aphp.fr (E.D.); aullene.toussaint@curie.fr (A.T.); enora.laas@curie.fr (E.L.); jean-guillaume.feron@curie.fr (J.-G.F.); virginie.fourchotte@curie.fr (V.F.); fabrice.lecuru@curie.fr (F.L.); noemie.girard@curie.fr (N.G.); 3 Data Factory, Institut Curie, Université Paris, 75005 Paris, France; amyn.kassara@curie.fr (A.K.); julien.guerin@curie.fr (J.G.); 4 Department of Medical Oncology, Institut Curie, Université Paris, 75005 Paris, France; florence.coussy@curie.fr (F.C.); beatrice.lavielle@curie.fr (B.L.); jean-yves.pierga@curie.fr (J.-Y.P.); anne-sophie.hamy-petit@curie.fr (A.-S.H.); 5 Department of Anesthesiology, Institut Curie, Université Paris, 75005 Paris, France; irene.kriegel@curie.fr; 6 Department of Radiotherapy, Institut Curie, Université Paris, 75005 Paris, France; youlia.kirova@curie.fr; 7 Residual Tumor & Response to Treatment Laboratory, RT2Lab, INSERM, U932 Immunity and Cancer, Institut Curie, Université Paris, 75005 Paris, France

**Keywords:** breast cancer, smoking status, smoking cessation, tobacco

## Abstract

**Simple Summary:**

There is little available evidence concerning smoking behaviors among breast cancer (BC) patients. This large study addresses smoking-related issues at BC diagnosis and smoking cessation in women with a history of BC. This study suggests that (i) tobacco mention is missing from electronic health records in approximately one-third of patients; (ii) tobacco is not assessed nor addressed systematically during the BC care pathway, and this information depends on the practitioner’s specialty; (iii) approximately one-third of patients stop smoking in BC follow-up. These findings call to consider BC treatment and follow-up as a window of opportunity to promote smoking cessation.

**Abstract:**

Breast cancer (BC) is the most commonly diagnosed type of cancer and the leading cause of cancer deaths in women. Smoking is the principal modifiable risk factor for cancers and has a negative influence on long-term survival. We conducted a retrospective study on consecutive BC survivors seen at follow-up consultations between 3 June and 30 October 2019 at Institut Curie, Paris, France. Smoking behaviors were evaluated prospectively via interviewer-administered questionnaires. The aim of this study was to describe smoking-related patient care at diagnosis and smoking cessation patterns in women with a history of BC. A total of 1234 patients were included in the study. Smoking status at diagnosis was missing from electronic health records in 32% of cases, including 13% of patients who smoke. Only 20% of the 197 patients currently smoking at diagnosis recalled having a discussion about smoking with a healthcare professional. Radiotherapists and surgeons were more likely to talk about complications than other practitioners. The main type of information provided was general advice to stop smoking (*n* = 110), followed by treatment complications (*n* = 48), while only five patients were referred to tobaccologists. Since diagnosis, 33% (*n* = 65) of the patients currently smoking had quit. Patients who quit had a lower alcohol consumption, but no other factor was associated with smoking cessation. The main motivation for tobacco withdrawal was the fear of BC relapse (63%). This study highlights room for improvement in the assessment of smoking behavior. Our data raise important perspectives for considering BC treatment and follow-up as a window of opportunity for smoking cessation.

## 1. Introduction

Breast cancer (BC) is a public health problem worldwide, with an estimated 2.1 million new cases and 627,000 deaths from BC in 2018 [1]. It is the most commonly diagnosed type of cancer and the leading cause of cancer deaths in women worldwide.

Tobacco use is the largest preventable cause of cancer worldwide, accounting for about 22% of all cancer-related deaths [2]. Causal relationships have been found between tobacco smoking and at least 20 types of cancer [3], including cancers of the lung, oral and nasal cavities, nasopharynx, oropharynx, hypopharynx, larynx, stomach, pancreas, colorectum, liver, kidney, ureter, bladder, cervix, and ovary, and acute myeloid leukemia. Conflicting findings have been reported concerning the link between smoking and a possible increase in the risk of BC [4]. However, recent studies, systematic reviews, and meta-analyses have concluded that there is a positive association between current smoking and BC incidence [5,6,7,8,9,10,11]. In 2016, Gram et al. [8] reported that one in six BC cases in patients who smoke could have been avoided if the patients concerned had not been actively smoking. This association may depend on smoking duration, lifetime exposure to tobacco, or the age at which the patient began smoking [5,7,9,10]. Passive smoking also seems to increase BC significantly, albeit to a lesser extent than active smoking [5,7,8,10,12].

Tobacco use in patients diagnosed with BC is associated with poor medical outcomes. Patients who currently smoke have a higher risk of regional BC spread at diagnosis than patients who never smoked (RR, 1.22; 95% CI 1.07 to 1.39, *p* = 0.005) [13]. Smoking is also responsible for postoperative complications, particularly after reconstructive surgery [14], radiation-induced toxicity [15], cardiovascular disease [16], and a poorer overall quality of life [17]. Some authors have reported evidence of a higher risk of recurrence [18], contralateral disease [19], and second primary cancer [20] in women with BC who smoke. Cigarette consumption and chest radiation increase the risk of ipsilateral lung cancer synergically in BC patients [21]. Finally, several studies [19,22,23,24,25] and meta-analyses [26,27] have shown that smoking increases the risk of all-cause and BC-specific mortality.

Surprisingly, little is known about the impact on survival outcomes of stopping smoking after BC diagnosis. Between 4% and 45% of female patients smoking quit after being diagnosed with BC [25,26,28,29,30,31]. Passarelli et al. [25] evaluated the impact of smoking cessation on 20,691 BC survivors. In this well-powered study, smoking cessation was associated with a 33% decrease in BC-specific mortality and a 9% decrease in all-cause mortality relative to the patients who did not quit smoking. This difference was accounted for by a statistically significant 60% decrease in the risk of death from respiratory cancer and a 20% lower risk of death from cardiovascular disease, in particular. In a retrospective study, Jizzini et al. [30] confirmed that smoking cessation was associated with better survival in a cohort of 31,069 BC survivors.

There is little available evidence concerning the prevalence of specific smoking cessation behaviors among BC survivors. In this study, we investigated the prevalence of smoking, and of assessments and counseling at BC diagnosis, the prevalence of smoking cessation after BC diagnosis, and the reasons for quitting, and the methods used by patients to quit smoking.

## 2. Materials and Methods

### 2.1. Patients

We prospectively included 1234 consecutive female patients attending follow-up consultations after surgery for BC in the breast and gynecological surgery department of Institut Curie (Paris, France) between 3 June and 30 October 2019. The study was approved by the Breast Cancer Study Group of Institut Curie and was conducted in accordance with institutional and ethical rules regarding research on patients. All participants provided verbal informed consent before inclusion. The French regulations did not require written informed consent from the patients for this study.

### 2.2. BC Treatments and Healthcare Providers

Patients were treated in accordance with national guidelines. All the patients were initially referred to a surgeon and saw an anesthesiologist as part of their care pathway. Patients treated by chemotherapy had a consultation with a medical oncologist, and patients treated by radiation therapy had a consultation with a radiotherapist. Follow-up involved twice-yearly consultations for at least five years, either systematically at the cancer center or via alternate outpatient and cancer center appointments. The patient’s pathway from BC diagnosis to follow-up consultation is described in Supplemental Figure S1.

### 2.3. Data Collection and Smoking Survey

The characteristics of the patients, tumors, and treatments at BC diagnosis were extracted from electronic health records (EHR).

Additional characteristics were assessed prospectively, with interviewer-administered questionnaires (Figure S2), during follow-up consultations with two medical residents (MN and ED). Patients were asked about their lifestyles: alcohol consumption (number of drinks per week), cannabis use (never/current/former user), physical activity (more or less than 30 min per day), and smoking profile (smoking history, smoking status at diagnosis and during follow-up, smoking intensity (mean number of cigarettes smoked per day), smoking duration (age at initiation, total number of years as a smoker)).

Patients who used to smoke were defined as women who had smoked at least 100 cigarettes in their lifetime but were not currently smoking at the time of BC diagnosis. Patients who currently smoke were defined as women who were actively smoking at the time of BC diagnosis. All other women were classified as patients who never smoked. Smoking status was determined both at BC diagnosis and at the time of inclusion in the study.

Women who were currently smoking at BC diagnosis were specifically asked questions about the assessment of their tobacco use at BC diagnosis. These questions concerned the inquiries relating to smoking status made by the healthcare professional at the time. The women were asked whether the healthcare professional had delivered any information regarding current tobacco use: (i) advice to quit or information about the general benefits of giving up smoking (including effects on all-cause mortality, cardiovascular and respiratory risks, and economic benefits); (ii) information about BC treatment-related complications induced by smoking (i.e., at least item of information about at least one of the following complications: secondary cancer, recurrence, BC-specific mortality, post-surgery infection, radiation therapy toxicity); (iii) assistance and advice about quitting methods (including, in particular, referral to a smoking cessation specialist). These questions were repeated for each of the healthcare professionals encountered by the patient during her care pathway (surgeon, anesthesiologist, oncologist, and radiotherapist).

Patients who stopped smoking after BC diagnosis were asked about their reasons for quitting, using a prespecified list of possible reasons (fear of recurrence or of other cancer/fear of complications/desire for breast reconstruction), the method they had used to stop smoking, and the total time for which they had stopped smoking since BC diagnosis.

### 2.4. Statistical Analysis

The study population is described in terms of frequencies for qualitative variables or medians and associated ranges for quantitative variables. To compare continuous variables among different groups, Wilcoxon–Mann–Whitney test was used for groups including less than 30 patients and for variables displaying multimodal distributions; otherwise, we used Student’s *t*-test.

Association between categorical variables was assessed with the chi-square test or with the Fisher’s exact test if at least one category included less than three patients. In boxplots, lower and upper bars represent the first and third quartile, respectively, the medium bar is the median, and whiskers extend to 1.5 times the inter-quartile range.

Data were processed, and statistical analyses were performed with R software version 3.1.2 (www.cran.r-project.org, accessed date: 2 January 2021, R Foundation for Statistical Computing, 2009).

### 2.5. Study Endpoints

The primary endpoint was smoking prevalence and the assessment of smoking habits and counseling provided by healthcare professionals at BC diagnosis. The secondary endpoints were the prevalence of smoking cessation after BC diagnosis, the reasons for quitting, and the methods used to quit.

## 3. Results

### 3.1. Baseline Characteristics of the Patients and Their Tumors

We included 1234 patients in the cohort (Table S1). The median age at BC diagnosis was 58 years, and 483 patients were overweight or obese (39.1%). BC subtype was as follows: luminal, *n* = 841 (85.2%), TNBC *n* = 69 (7%), *HER2*-positive, *n* = 77 (7.8%). All patients had undergone surgery, 80.4% had undergone radiotherapy, 25.5% chemotherapy, 64% endocrine therapy, and 10.2% had undergone breast reconstruction (immediately in 4.6% and secondary surgery in 5.6%).

### 3.2. Smoking at Diagnosis

**Figure 1:**
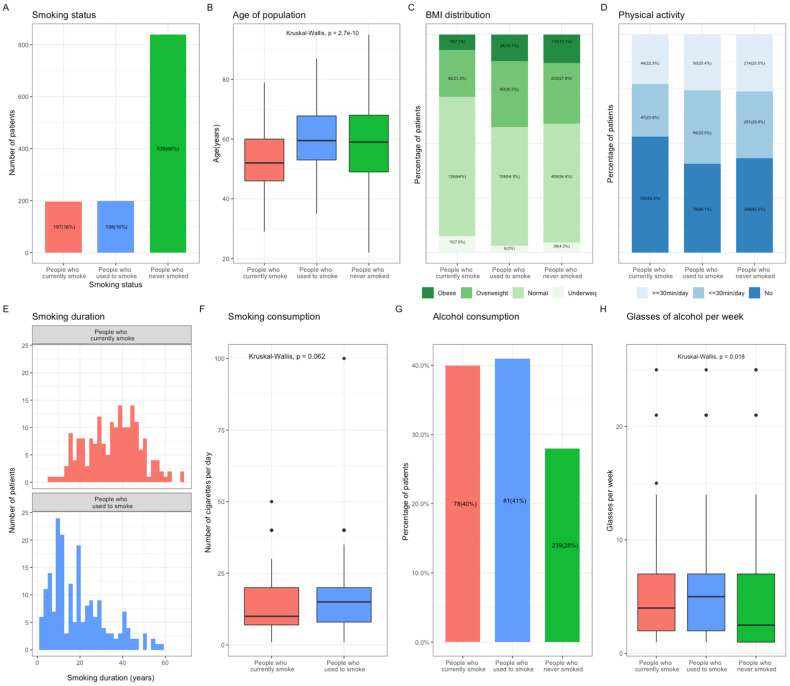
Population characteristics according to smoking status at the time of Breast Cancer (BC) diagnosis. (**A**), Repartition of the population according to smoking status at diagnosis. (**B**), Mean age of the population according to smoking status at diagnosis. (**C**), Bar plot representing the BMI distribution in the population according to smoking status at diagnosis. Subcategories were defined as followed: Obese, BMI > 30 kg/m^2^; Overweight, BMI > 25 kg/m^2^; Normal, 18 kg/m^2^ < BMI < 25 kg/m^2^; Underweight, BMI < 18 kg/m^2^. (**D**), Bar plot representing the physical activity in the population according to smoking status at diagnosis. Subcategories were defined as followed: Yes, more or less than 30 min per day; No. (**E**), Smoking duration in patients who currently smoke, and those who used to smoke at diagnosis. (**F**), Mean smoking consumption in patients who currently smoke and those who used to smoke at diagnosis. (**G**), Bar plot representing the patients’ alcohol consumption according to smoking status at diagnosis. (**H**), Mean number of alcohol glasses consumed per week according to smoking status at diagnosis. Abbreviation: BC, breast cancer; BMI, body mass index.

The distribution of smoking status at BC diagnosis was as follows: patients who currently smoke, *n* = 197 (16%), patients who used to smoke, *n* = 198 (16%), and patients who never smoked, *n* = 839 (68%) (Figure 1A). The patients who currently smoke were significantly younger than the patients who used to smoke or those who have never smoked (currently smoking: 52.7; used to smoke: 60.2; never smoked: 58.7, *p* < 0.001, Figure 1B and Figure S3). They were also more likely to be of normal weight or underweight (71.6%) than patients who used to smoke (57.5%) and those who have never smoked (59.1%) (Figure 1C, *p* < 0.001), and to have no physical activity at all (currently smoking: 52.3%; used to smoke: 40.4%; never smoked: 42.2%, Figure 1D). The median duration of smoking was 35.6 years in patients currently smoking, which was greater than that in patients who used to smoke (Figure 1E), although the total exposure to tobacco smoking in these two groups was similar (Figure 1F). The patients who never smoked drank alcohol both less frequently and in smaller quantities than those who currently or used to smoke (Figure 1G).

### 3.3. Assessment, by Healthcare Providers, of Smoking Habits at Diagnosis

**Figure 2:**
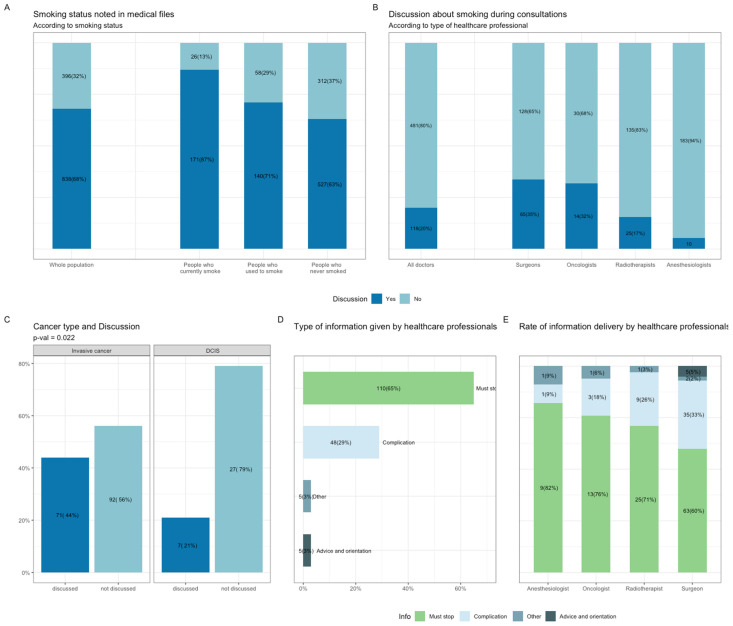
Smoking assessment and counseling by health care professionals at the time of BC diagnosis. (**A**), Smoking assessment reported in electronic health records (EHR) among the whole population and according to smoking status at diagnosis. (**B**), Smoking discussion during consultations with health care practitioners and according to each specialist (Surgeons, Oncologists, Radiotherapists, Anesthesiologists). (**C**), Discussion on tobacco consumption according to cancer type. (**D**), Information type provided by health care professionals. Subcategories were: general advice on smoking cessation (Must stop); treatment complications (Complication); practical advice and orientation (Advice and orientation) and Other. (**E**), Information type according to each specialist. Abbreviation: BC, breast cancer; EHR, electronic health records; BMI, body mass index.

Smoking status at BC diagnosis was missing from the EHR in 32% of cases, including 13% of patients who currently smoke (Figure 2A). For the population of patients currently smoking at BC diagnosis (*n* = 197), 20% of the patients recalled having a discussion about tobacco consumption with a healthcare professional (*n* = 118; total of 599 consultations with an anesthesiologist, medical radiotherapist, surgeon, or oncologist). Surgeons were the specialists most likely to raise the subject of tobacco consumption (35%), and anesthesiologists were the least likely to do so (6%) (Figure 2B). Type of cancer was the only patient or tumor characteristic significantly associated with the likelihood of discussing tobacco consumption (Table S2, Figure 2C). The information provided was principally general advice to stop smoking (*n* = 110, 65%), followed by information about treatment complications (*n* = 48, 29%), with only very few patients receiving practical advice about how to quit smoking or being referred to a smoking cessation specialist (Figure 2D). Radiotherapists and surgeons were more likely than the other healthcare professionals to talk about complications (Figure 2E).

### 3.4. Smoking Cessation After Diagnosis

**Figure 3:**
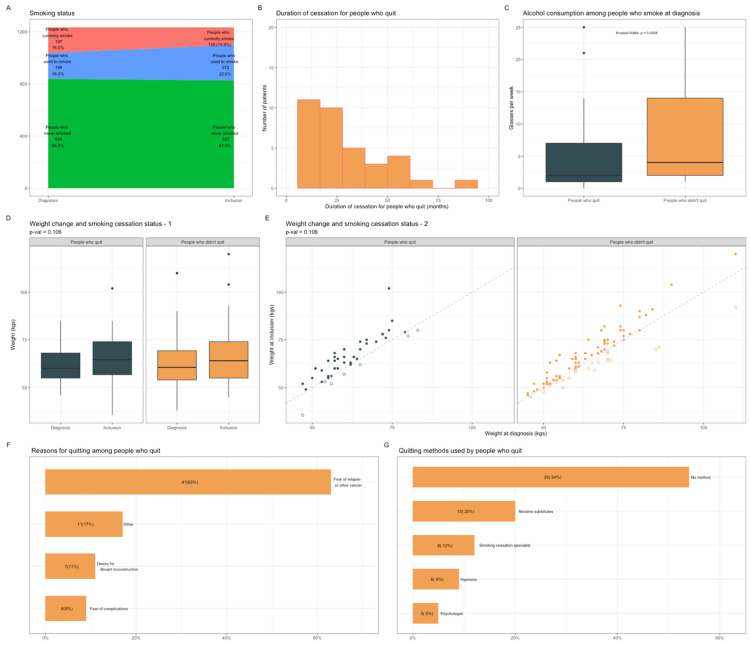
Population characteristics according to smoking cessation status after BC diagnosis. (**A**), Patients’ repartition according to smoking status at the time of diagnosis and at inclusion. (**B**), Smoking cessation duration among patients who quit smoking (*n* = 65). (**C**), Alcohol consumption among patients who currently smoke at diagnosis according to smoking cessation status. (**D**), Mean weight changes between diagnosis and study inclusion according to smoking cessation status. (**E**), Scatter plot representing weight changes since BC diagnosis according to smoking cessation status after diagnosis. Full circles representing patients who have gained weight; empty ones, patients who have lost weight. (**F**), Smoking cessation motivations among patients who quit smoking (*n* = 65). Prespecified subcategories were: Fear of relapse or other cancer; Desire of a healthier life (Other); Desire of breast reconstruction and Fear of complications. (**G**), Smoking cessation methods among patients who quit smoking (*n* = 65). Subcategories were: No method; Nicotinic substitutes; Tobacco consultations; Alternative methods (Hypnosis); Consultation with a psychologist. Abbreviation: BC, breast cancer.

Overall, 33% (*n* = 65) of the patients who currently smoke at BC diagnosis had quit smoking after diagnosis (Figure 3A), for a median of 17.5 months (Figure 3B). The patients who quit had lower levels of alcohol consumption (2 glasses per week vs. 4, Figure 3C), but no other factor related to tumor characteristics or treatments received was associated with smoking cessation (Table S3). While the occurrence of the discussion was not associated with the duration of follow-up, smoking cessation was significantly associated with the duration of follow-up. This finding is consistent with the cumulative probability of a given individual being exposed to an event. In this vein, the long follow-up required in BC represents contact points to the healthcare system to reassess regularly smoking status and offer smoking cessation (Figures S4 and S5). Median weight increased between diagnosis and study inclusion (+2.7 kgs), but changes in weight did not differ significantly between the patients who quit and those who had not (Figure 3D,E), *p* = 0.106. The main reason for stopping smoking was fear of relapse (63%) (Figure 3F). Most of the patients who quit did not use any specific method for quitting (*n* = 35, 54%)), thirteen used nicotine substitutes, eight consulted a smoking cessation specialist, six used hypnosis, and three had psychological support (Figure 3G).

## 4. Discussion

In this large study, we found that approximately one-third of the BC patients who were currently smoking at diagnosis subsequently stopped smoking. These results are of importance because, unlike most tobacco-induced cancers, such as lung and head and neck cancers, BC is a highly curable disease. Thus, 90% of BC survivors may have genuinely cured cancer, but they remain at a higher risk of death than the general population if they continue to smoke. Our study provides insight into smoking-related issues in the BC field.

First, the prevalence of active smoking at diagnosis was 16%. Several studies have evaluated smoking status at diagnosis and found that 7.5% to 25% of patients with BC are actively smoking [6,7,14,20,21,24,25,26,28,29,30]. Our results are consistent with these findings, but the prevalence of active smoking among our patients at diagnosis was lower than that among women generally in France, which has been estimated at 26.4% [32]. However, this is possibly due to the median age of our cohort (58 years old). Indeed, the prevalence of smoking decreased with age, and frequencies of active smoking in our cohort were in line with published results when we performed age-specific comparisons [32].

Second, we found that tobacco use was not systematically assessed or addressed during the BC care pathway. One-third of the medical records did not document smoking status at diagnosis, even for a significant portion of patients currently smoking (13.2%). Peters et al. [33] showed that only 29% of U.S National Cancer Institute Cooperative Group clinical trials assessed tobacco use status at enrollment. In addition, 80% of healthcare providers failed to provide their patients with any information on smoking during the course of treatment for BC. In a study conducted by the French Cancer Institute (INCa) in 2014 [34], on 1076 healthcare professionals, 80% said that they discussed smoking with cancer patients, and 90% considered the promotion of smoking cessation to be a part of their role. Our results indicate that information is delivered less frequently in routine practice, or at least that the patient’s perception is that information delivery is less frequent. Several potential barriers might deter healthcare professionals from advising cancer survivors to quit. Such barriers include a lack of knowledge about how to assess tobacco use and dependence, or about the efficacy of treatment, time constraints, or too low a priority being accorded to the importance of smoking cessation when treating cancer patients [35,36,37]. Physicians may also have concerns about their patients’ potential reactions, lack of motivation, or may fear exacerbating the guilt and shame that smokers often feel after they have developed cancer.

Our study also highlighted the general nature of the information provided, with a lack of specific advice concerning smoking-related complications after BC diagnosis. The quality of information is important because current smokers with BC may be more motivated to quit smoking if healthcare professionals explain the increase in BC-specific risks associated with smoking in clear, strong, personalized terms rather than providing general information about smoking.

The 3.1% rate of referral to specialists was low. In a study on 74 healthcare professionals, fewer than 30% reported referring smoking patients to specialists in smoking cessation [35]. Similarly, in the VICAN 5 study, one in four patients reported having been offered a consultation with a specialist [38]. Our results indicate that there is room for improvement, as referral to a specialist is a useful action. Nolan et al. implemented an intervention in which all breast cancer patients who smoked were referred to smoking cessation services [39]. The proportion of smoking patients referred to a specialist in smoking cessation increased from 29% (22/75) before the intervention to 74% (20/27) afterward. Attendance at the consultation increased from 41% (9/22) before the intervention to 75% (15/20) after the intervention. This finding is particularly important, as a patient who smokes, wants to quit, and receives help has an 80% higher chance of success than a patient who quits without assistance [40].

Third, the rate of smoking cessation after BC diagnosis was 33%, a rate higher than that for the general population but similar to that reported in other studies [25,26,28,29,30,31,38], ranging from 4% to 45%. In a study based on the follow-up of more than 12,000 smokers with no prior diagnosis of cancer, the rate of smoking cessation was higher among those diagnosed with cancer during the follow-up period than among those with no such diagnosis (31% vs. 19%) [31]. This finding suggests that cancer diagnosis constitutes a moment at which the patient is receptive to information and should be considered as a unique opportunity to encourage the patient to stop smoking. Previous studies found that patients diagnosed with BC were more likely to continue smoking than patients with lung, head, or neck cancers, suggesting that the perception of causality between tobacco and the type of cancer plays a role in cessation [38,41]. Efforts should therefore be made to explain the additional benefits of stopping smoking. In the VICAN 5 study, the patients who considered the possibility of relapse were found to be more at risk of continuing to smoke than those who did not (19.3% vs. 14.7%). By contrast, the top reason for quitting in our study was the fear of relapse or other cancer, well ahead of the fear of complications and a desire for reconstruction surgery.

Finally, our study suggests that surgical treatment represents an opportunity to encourage patients to stop smoking. The French High Authority for Health (HAS) recommends routine screening for smoking before any surgery and the suggestion that the patient should stop smoking, or at least use nicotine substitutes to reduce smoking levels, at least six weeks before surgery [42]. In our study, surgeons were the healthcare professionals most likely to provide patients with information about smoking, possibly because they will have to cope with smoking-related surgical complications. Thomsen et al. assessed the efficacy of nicotine replacement therapy given perioperatively, with a single motivational counseling session, to patients diagnosed with BC [43]. The authors detected a significant difference in abstinence rates between the intervention and usual care groups (28% vs. 11%) 10 days after surgery, but this difference was no longer significant after 12 months. In a meta-analysis on 13 trials, Nayan and coworkers [44] showed that the perioperative period was associated with more than double the odds of quitting (OR = 2.31 (95% CI, 1.32–4.07)) relative to clinic-based enrollment.

Our study has several strengths. This is, to our knowledge, the largest study addressing smoking-related issues at BC diagnosis and smoking cessation patterns in women with a history of BC. It also has limitations. Smoking status was self-reported, and we cannot exclude the possibility that cancer survivors may misrepresent their smoking status [45]. Finally, there may be a negative recall bias concerning the reception of information about smoking at diagnosis, and this bias may be stronger among patients who fail to quit smoking.

Our results open up several perspectives. They support further improvements in the assessment of smoking behavior by healthcare providers. One useful tool for the systematic assessment of smoking status would be the inclusion of structured, prospective, standardized, evidence-based smoking assessments in the EHR at the time of diagnosis and periodically during follow-up. They also highlight the need to improve the training of healthcare providers, their knowledge about how to approach the issue of smoking cessation with cancer patients, and the need for protocols to guide their practice. The organization of the care pathways for cancer patients should also include the availability of information documents for delivery to patients. The AAC approach—ask, advise, connect—[46]—is also based on a local network to guide the patient and referral to cessation programs within treatment facilities, which should be incorporated into supportive care.

The active phase of BC treatment requires multiple visits to the cancer center, which could be seen as points of contact with the healthcare system. There is evidence to suggest that rates of smoking cessation are higher if support to quit smoking is offered at the time of cancer diagnosis, resulting in higher success rates and a shorter time between diagnosis and the initiation of a quitting program [47]. Finally, after treatment completion, the follow-up phase of BC also facilitates phase-repeated counseling, which is a key feature for successfully quitting smoking.

## 5. Conclusions

Taking into account the well-known benefits of stopping smoking for wound healing, quality of life, and overall survival, BC treatment, and follow-up should be considered as a window of opportunity for addressing the patient’s tobacco use and provide guidance to help the patient to quit smoking effectively.

## Data Availability

Data available on request due to privacy/ethical restrictions. The data that support the findings of this study are available on request from the corresponding author [FR]. The data are not publicly available because they contain information that could compromise the privacy of research participants.

## Supplementary Materials

The following are available online at https://www.mdpi.com/article/10.3390/cancers13102423/s1; Figure S1: The patient’s pathway since BC diagnosis to follow-up consultation, Figure S2: interviewer-administered questionnaires based on a codebook, Figure S3: Smoking status according to age classes, Figure S4: Patient follow-up durations according to smoking cessation status after diagnosis, Figure S5: Patient follow-up durations according to the likelihood of discussing tobacco consumption in follow-up consultations, Table S1: Patient’s characteristics among the whole population and according to tobacco status at the time of BC diagnosis, Table S2: Characteristics of patients who currently smoke according to smoking assessment by healthcare professionals at BC diagnosis, Table S3: Characteristics of patients who currently smoke at BC diagnosis according to smoking cessation status.
