# Supplementary material for: Breast Cancer (BC) Is a Window of Opportunity for Smoking Cessation: Results of a Retrospective Analysis of 1234 BC Survivors in Follow-Up Consultation
Source: Cancers (Basel). 2021 May 17;13(10):2423. doi: 10.3390/cancers13102423 (PMC8156674; doi:10.3390/cancers13102423)
Supplement: Supplementary file 1 [file cancers-13-02423-s001.zip › cancers-1184868-supplementary.pdf]

Supplementary Materials

Breast Cancer (BC) Is a Window of Opportunity for Smoking Cessation: Results of a Retrospective Analysis of 1234 BC Survivors in Follow-Up Consultation

Marion Nicolas, Beatriz Grandal, Emma Dubost, Amyn Kassara, Julien Guerin, Aullene Toussaint, Enora Laas, Jean-Guillaume Feron, Virginie Fourchotte, Fabrice Lecuru, Noemie Girard, Florence Coussy, Beatrice Lavielle, Irene Kriegel, Youlia Kirova, Jean-Yves Pierga, Fabien Reyal and Anne-Sophie Hamy

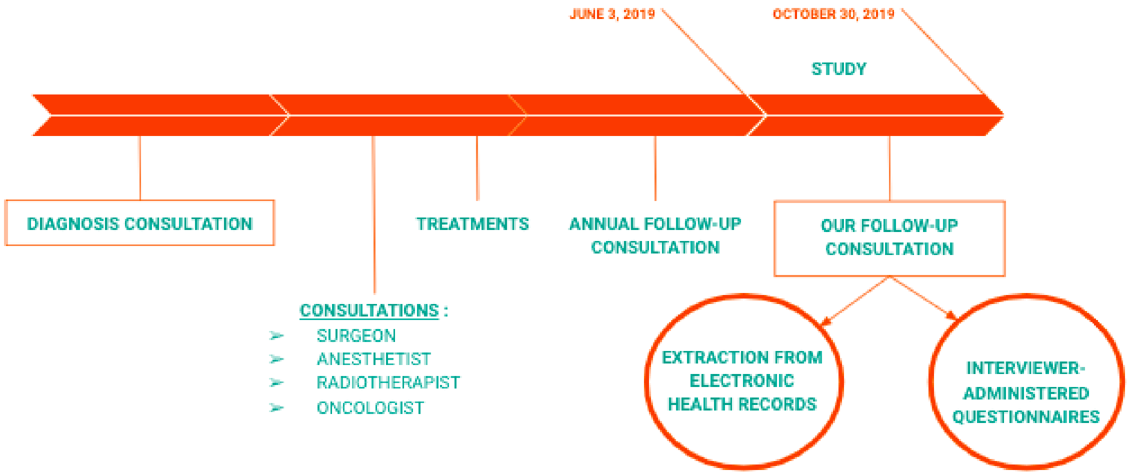

Figure S1. The patient's pathway since breast cancer diagnosis to follow-up consultation.

Tabac PID 134

Codebook

Data Dictionary Codebook

03/05/2021 09:39

Collapse all instruments

#	Variable / Field Name	Field Label<br>*Field Note*	Field Attributes (Field Type, Validation, Choices, Calculations, etc.)
Instrument: **Questionnaire Tabac** (questionnaire_tabac)			Collapse
1	record_id	IPP	text, Identifier
2	date_consult	Consultation day	text (date_dmy, Min: 2019-05-01), Required
3	dat_birth	Birth date	text (date_dmy, Min: 1920-01-01)
4	habitus	Habitus	notes
5	niv_etu	Highest educationnal level	dropdown<br>1 never been enrolled<br>2 elementary school<br>3 no degree but enrolled until after elementary school<br>4 primary school certificicate<br>5 college diploma<br>6 certificate of professional competence<br>7 professional teaching certificate<br>8 general baccalaureate<br>9 technological baccalaureate<br>10 first degree diploma<br>11 second or third degree diploma
6	csp	Profession at diagnosis	dropdown<br>1 Agriculture, forestry, fishing<br>2 Energy Industry<br>3 Others industries<br>4 Construction<br>5 Trade<br>6 Education, health, social work<br>7 Other service<br>8 None<br>9 Retired
7	age_at_surg	Age	calc<br>Calculation: round(datediff([date_chir],[dat_birth],"y","dmy"),0)
8	weight	Weight	text (number, Min: 35, Max: 190)
9	size	Size	text (number, Min: 100, Max: 210)
10	stat_mar	Marital status	dropdown<br>1 single<br>2 couple<br>3 married<br>4 divorced<br>5 widow
11	tobac_mention	Tobacco mentionned in the medical report	yesno<br>1 Yes<br>0 No
12	tabac_life	Tobacco (at least 100 cig in life)	yesno, Required<br>1 Yes<br>0 No

	13	smoking	Currently smoking	yesno<br>1 Yes<br>0 No
	14	tobac_diag<br>Show the field ONLY if:<br>[tabac_life] = '1'	Tobacco at diagnosis	yesno<br>1 Yes<br>0 No
	15	age_tobac_start<br>Show the field ONLY if:<br>[tabac_life] = '1'	Age of tobacco beginning	text (integer, Min: 8, Max: 80)
	16	tobac_years<br>Show the field ONLY if:<br>[tabac_life] = '1'	Number of year of smoking	text (integer)
	17	nb_cig_life<br>Show the field ONLY if:<br>[tabac_life] = '1'	Average number of cig per day	text (integer, Min: 1, Max: 100)
	18	age_tobac_stop<br>Show the field ONLY if:<br>[tobac_diag] = '0'	Age at smoking cessation	text (integer, Min: 10, Max: 100)
	19	recent_smoker<br>Show the field ONLY if:<br>[tobac_diag] = '0'	Smoking cessationg during the year before diagnosis	yesno<br>1 Yes<br>0 No
	20	alcool	Alcohol (nb glasses / week)	text (integer, Min: 0, Max: 25)
	21	drogue	Drugs (cannabis, others, etc)	dropdown<br>0 currently<br>1 ever<br>2 never
	22	comm_drogue<br>Show the field ONLY if:<br>[drogue] = '0' or [drogue] = '1'	Comments about drugs	notes
	23	sport	Physical activity	yesno<br>1 Yes<br>0 No
	24	comm_sport<br>Show the field ONLY if:<br>[sport] = '1'	Duration of daily physical activity	checkbox<br>1 comm_sport___1 < 30 min/d<br>2 comm_sport___2 > 30 min/d
	25	in_situ_exclus	In situ exclusive carcinoma	yesno<br>1 Yes<br>0 No
	26	statut_tn	Clinical status	text
	27	type_histo<br>Show the field ONLY if:<br>[in_situ_exclus] = '0'	Histological type	checkbox<br>0 type_histo___0 CCI<br>1 type_histo___1 CLI<br>2 type_histo___2 CCIS<br>3 type_histo___3 CLIS<br>4 type_histo___4 Others
	28	bifocal	Multifocal	yesno<br>1 Yes<br>0 No
	29	rec_est<br>Show the field ONLY if:<br>[in_situ_exclus] = '0'	Estrogen receptors	checkbox<br>0 rec_est___0 positive<br>1 rec_est___1 negative<br>2 rec_est___2 na

	30	recep_prog<br>Show the field ONLY if:<br>[in_situ_exclus] = '0'	Progesterone receptors	checkbox<br>0 | recep_prog___0 | positive<br>1 | recep_prog___1 | negative<br>2 | recep_prog___2 | na
	31	stat_her2<br>Show the field ONLY if:<br>[in_situ_exclus] = '0'	Her2 status	checkbox<br>0 | stat_her2___0 | positive<br>1 | stat_her2___1 | negative<br>2 | stat_her2___2 | na
	32	grade<br>Show the field ONLY if:<br>[in_situ_exclus] = '0'	Histological grade	checkbox<br>0 | grade___0 | 1<br>1 | grade___1 | 2<br>2 | grade___2 | 3<br>3 | grade___3 | na
	33	embole<br>Show the field ONLY if:<br>[in_situ_exclus] = '0'	Embolus	checkbox<br>0 | embole___0 | yes<br>1 | embole___1 | no<br>2 | embole___2 | na
	34	nb_gg<br>Show the field ONLY if:<br>[in_situ_exclus] = '0'	Number of positive nodes	text (integer, Min: 0, Max: 100)
	35	ttt_kr	Treatment type	checkbox<br>0 | ttt_kr___0 | surgery<br>1 | ttt_kr___1 | chemotherapy<br>2 | ttt_kr___2 | radiotherapy<br>3 | ttt_kr___3 | trastuzumab<br>4 | ttt_kr___4 | endocrine therapy<br>5 | ttt_kr___5 | breast reconstruction
	36	hormono_neo<br>Show the field ONLY if:<br>[ttt_kr(4)] = '1'	Neoadjuvant endocrine therapy	yesno<br>1 | Yes<br>0 | No
	37	date_hormono<br>Show the field ONLY if:<br>[hormono_neo] = '1'	Start date for neoadjuvant endocrine therapy	text (date_dmy)
	38	seq_chimio<br>Show the field ONLY if:<br>[ttt_kr(1)] = '1'	Chemotherapy sequence	dropdown<br>0 | neoadjuvant<br>1 | adjuvant<br>2 | neoadjuvant and adjuvant
	39	date_chimio<br>Show the field ONLY if:<br>[seq_chimio] = '0'	Start date for neoadjuvant chemotherapy	text (date_dmy)
	40	taille_infiltrant<br>Show the field ONLY if:<br>[ttt_kr(0)] = '1' or [ttt_kr(2)] = '1' or [ttt_kr(3)] = '1' or [ttt_kr(4)] = '1' or [seq_chimio] = '1'	Tumor size (mm)	text (integer, Min: 0, Max: 250)
	41	reponse_cna<br>Show the field ONLY if:<br>[seq_chimio] = '0' or [seq_chimio] = '2'	Neoadjuvant chemotherapy result	dropdown<br>0 | pCR with in situ remaining<br>1 | pCR (pathological complete response)<br>2 | no pCR
	42	chir<br>Show the field ONLY if:<br>[ttt_kr(0)] = '1'	Surgery type	dropdown<br>0 | lumpectomy<br>1 | mastectomy<br>2 | immediate breast reconstruction

	43	date_chir<br><br>Show the field ONLY if:<br>[ttt_kr(0)] = '1'	Surgery date	text (date_dmy, Min: 1960-01-01)
	44	reconst<br><br>Show the field ONLY if:<br>[chir] = '1'	Postponed breast reconstruction	yesno<br>1 Yes<br>0 No
	45	anes_tabac<br><br>Show the field ONLY if:<br>[tabac_diag] = '1' and [ttt_kr(0)] = '1'	Smoking status asked by anesthesiologist	yesno<br>1 Yes<br>0 No
	46	info_tabac_anes<br><br>Show the field ONLY if:<br>[anes_tabac] = '1'	Info smoking anesth	checkbox<br>0 info_tabac_anes___0 info complications<br>1 info_tabac_anes___1 info cessation needed<br>2 info_tabac_anes___2 advises and orientation
	47	chir_tabac<br><br>Show the field ONLY if:<br>[tabac_diag] = '1' and [ttt_kr(0)] = '1'	Smoking status asked by surgeon	yesno<br>1 Yes<br>0 No
	48	info_tabac_chir<br><br>Show the field ONLY if:<br>[chir_tabac] = '1'	info smoking surgeon	checkbox<br>0 info_tabac_chir___0 info complications<br>1 info_tabac_chir___1 info cessation needed<br>2 info_tabac_chir___2 advises and orientation
	49	rt_tabac<br><br>Show the field ONLY if:<br>[ttt_kr(2)] = '1' and [tabac_diag] = '1'	Smoking status asked by radiotherapist	yesno<br>1 Yes<br>0 No
	50	info_tabac_rt<br><br>Show the field ONLY if:<br>[rt_tabac] = '1'	Info smoking RT	checkbox<br>0 info_tabac_rt___0 info complications<br>1 info_tabac_rt___1 info cessation needed<br>2 info_tabac_rt___2 advises and orientation
	51	onco_tabac<br><br>Show the field ONLY if:<br>([ttt_kr(1)] = '1' and [tabac_diag] = '1' or [ttt_kr(3)] = '1' and [tabac_diag] = '1')	Smoking status asked by oncologist	yesno<br>1 Yes<br>0 No
	52	info_tabac_onco<br><br>Show the field ONLY if:<br>[onco_tabac] = '1'	Info smoking onco	checkbox<br>0 info_tabac_onco___0 info complications<br>1 info_tabac_onco___1 info cessation needed<br>2 info_tabac_onco___2 advises and orientation
	53	moyen_sevrage<br><br>Show the field ONLY if:<br>[info_tabac_chir(2)] = '1' or [info_tabac_anes(2)] = '1' or [info_tabac_rt(2)] = '1' or [info_tabac_onco(2)] = '1'	Methods used for smoking cessation	checkbox<br>0 moyen_sevrage___0 tobacco consultation<br>1 moyen_sevrage___1 psychological support<br>2 moyen_sevrage___2 cognitive behavioral therapy<br>3 moyen_sevrage___3 nicotine substitutes<br>4 moyen_sevrage___4 pharmaco<br>5 moyen_sevrage___5 hypnosis<br>6 moyen_sevrage___6 acupuncture
	54	tabac_surv	Tobacco status	dropdown<br>0 current<br>1 cessation before diagnosis<br>2 cessation at diagnosis<br>3 never
	55	duree_sevrage<br><br>Show the field ONLY if:<br>[tabac_surv] = '2'	How many month without smoking since diagnosis	text (integer, Min: 1, Max: 200)

	56	nb_cig_surv<br><br>Show the field ONLY if:<br>[tabac_surv] = '0'	Average number of cig /d	text (integer, Min: 1, Max: 50)
	57	methode_sevrage<br><br>Show the field ONLY if:<br>[tabac_surv] = '2'	Methods used for smoking cessation	checkbox<br>0 methode_sevrage___0 tobacco consultation<br>1 methode_sevrage___1 psychological support<br>2 methode_sevrage___2 cognitive behavioral therapy<br>3 methode_sevrage___3 nicotine substitutes<br>4 methode_sevrage___4 pharmaco<br>5 methode_sevrage___5 hypnosis<br>6 methode_sevrage___6 acupuncture
	58	motivation_sevrage<br><br>Show the field ONLY if:<br>[tabac_surv] = '2'	Motivation for smoking cessation	checkbox<br>0 motivation_sevrage___0 recurrence or other cancer fear<br>1 motivation_sevrage___1 complications fear<br>2 motivation_sevrage___2 breast reconstruction desire<br>3 motivation_sevrage___3 other"
	59	autre_motivation_sevrage<br><br>Show the field ONLY if:<br>[motivation_sevrage(3)] = '1'	Other motivation for smoking cessation	notes
	60	recidiv_cancer	Recurrence after treatment	yesno<br>1 Yes<br>0 No
	61	poids_actuel	Actual weight	text (number, Min: 0, Max: 200)
	62	comment	Comments	notes
	63	questionnaire_tabac_complet e	Section Header: *Form Status*<br>Complete?	dropdown<br>0 Incomplete<br>1 Unverified<br>2 Complete

Figure S2. Interviewer-administered questionnaires based on a codebook.

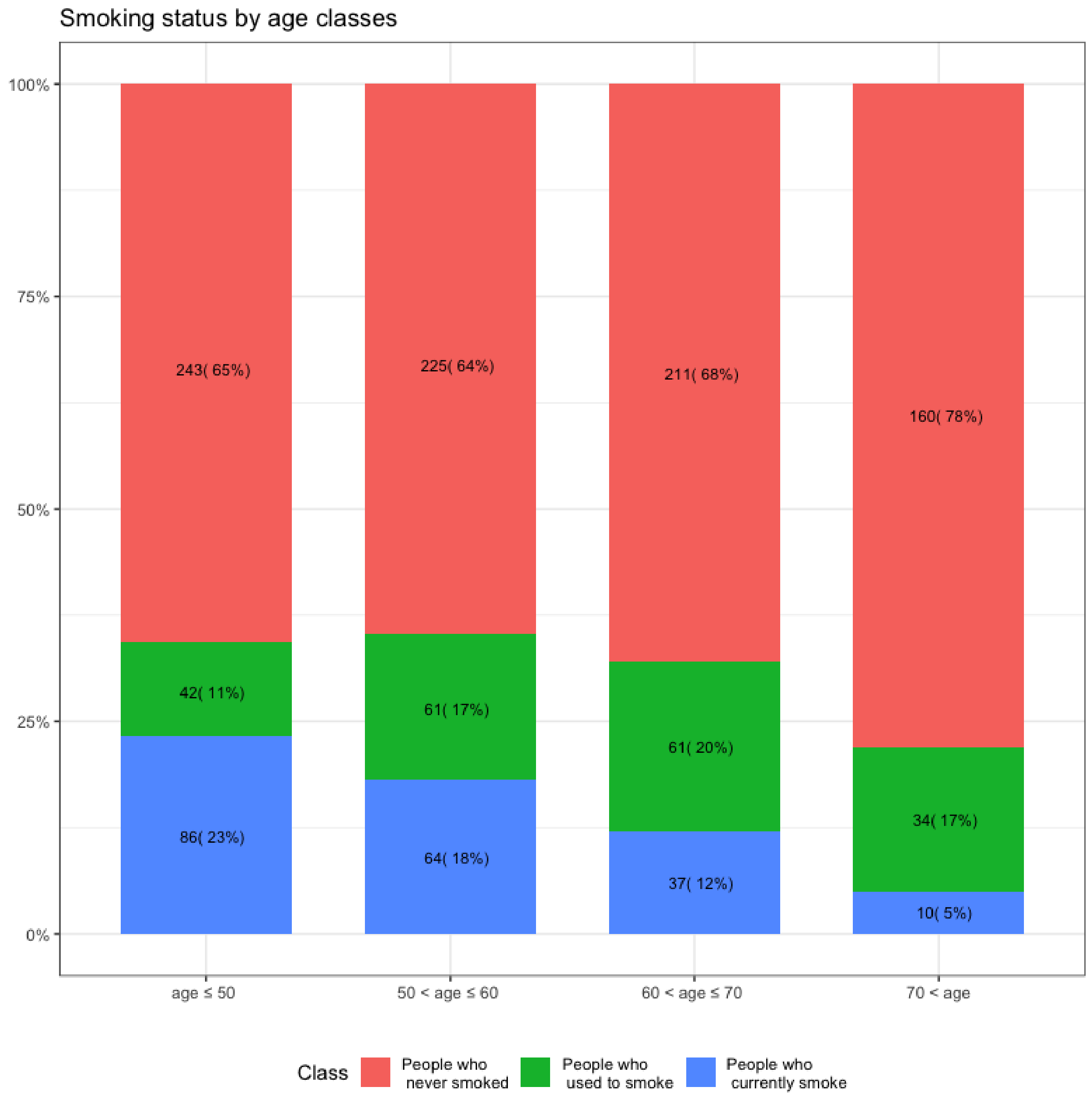

Figure S3. Smoking status according by age classes.

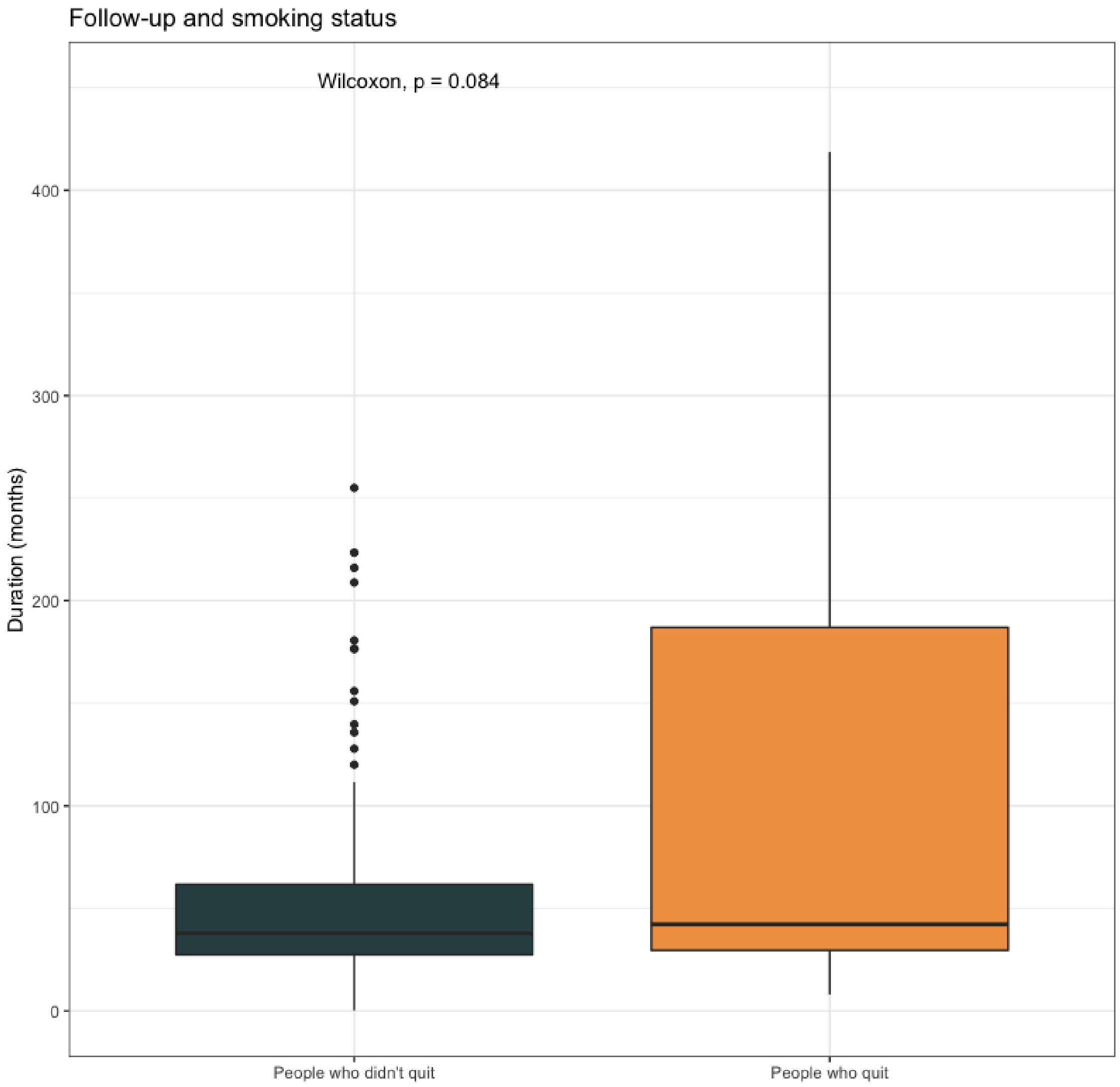

Figure S4. Patient follow-up durations according to smoking cessation status after diagnosis.

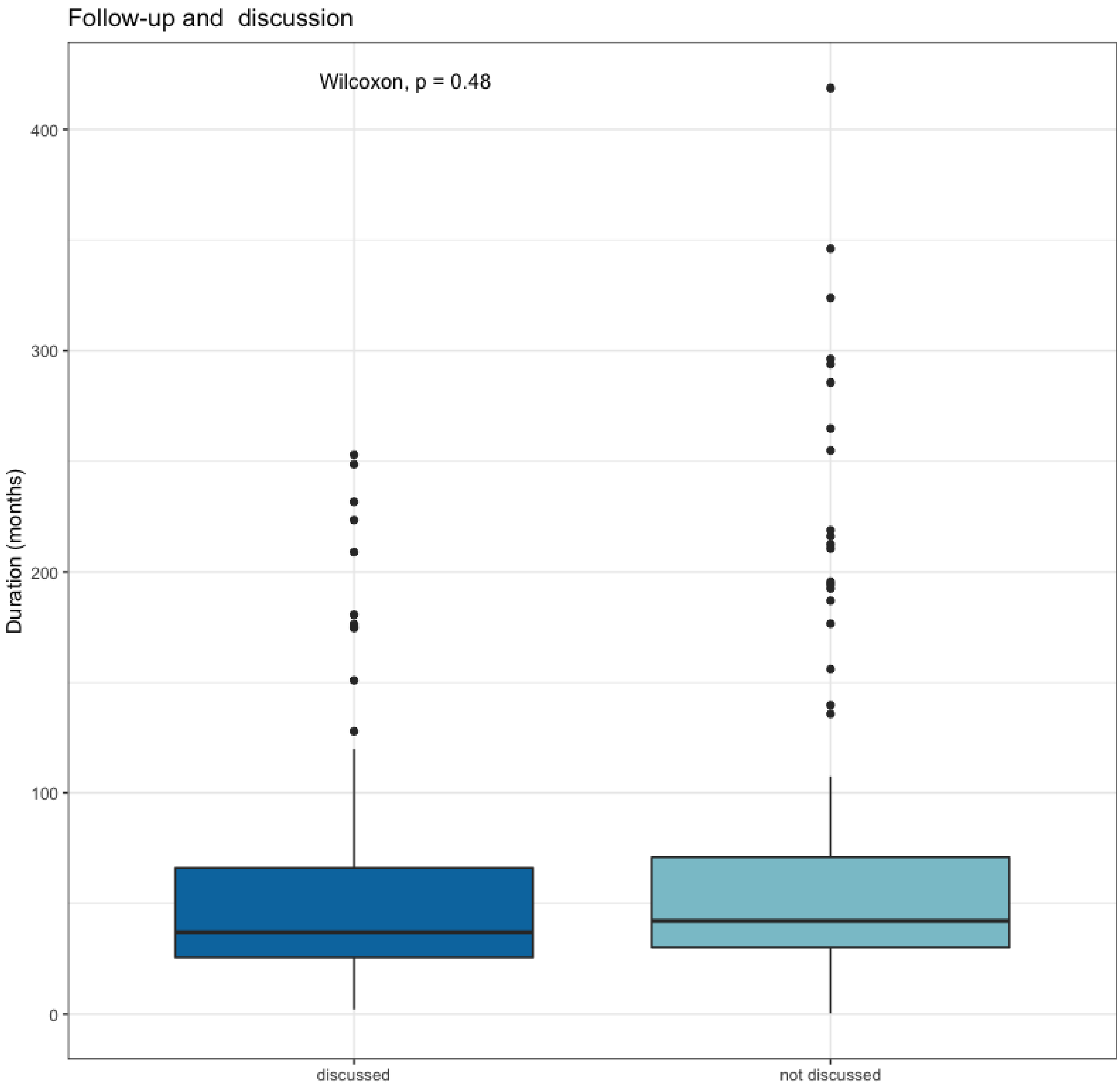

Figure S5. Patient follow-up durations according to the likelihood of discussing tobacco consumption in follow-up consultations.

Table S1. Patients' characteristics among the whole population and according to tobacco status at the time of BC diagnosis.

	Whole Population	Patients Who Currently Smoke	Patients Who Used To Smoke	Patients Who Never Smoked	*p*-Value
n	1234(100%)	197(16%)	198(16%)	839(68%)	
**Age** (years)	58.0 (12.2)	52.7 (10.4)	60.2 (10.9)	58.7 (12.6)	< 0 .001
**BMI** (kg/m2)	24.5 (4.7)	23.3 (4.2)	25.1 (4.7)	24.7 (4.8)	< 0 .001
Underweight	59(4.8%)	15(7.6%)	6(3.0%)	38(4.5%)	
Normal	692(56.0%)	126(64.0%)	108(54.5%)	458(54.6%)	
Overweight	335(27.1%)	42(21.3%)	60(30.3%)	233(27.8%)	
Obese	148(12.0%)	14(7.1%)	24(12.1%)	110(13.1%)	
**Smoking patterns**					
Age at onset	18 [17.0, 20.0]	18 [17.0, 20.0]	18.0 [16.0, 21.0]	None	0.413
Duration	27.4 (15.2)	35.6 (12.7)	19.5 (13.1)	None	<0.001
Amount (cig/day)	13.8 (9.6)	12.8 (8.0)	14.9 (10.8)	None	0.062
**Alcohol consumption**					< 0 .001
Yes	398(32.3%)	78(39.6%)	81(40.9%)	239(28.5%)	
Amount (glass/week)	5.3(5.1)	6.2(5.8)	6.3(5.9)	4.6(4.6)	
No	836(67.7%)	119(60.4%)	117(59.1%)	600(71.4%)	
**Drugs** (Cannabis or other)					< 0 .001
Current	4(0.3%)	4(2.1%)	0(0.0%)	0(0.0%)	
Ever	12(1.0%)	5(2.6%)	5(2.6%)	2(0.2%)	
Never	1211(98.7%)	186(95.4%)	191(97.4%)	834(99.8%)	
**Physical activity**					0.022
Yes	672(54.5%)	91(46.2%)	116(58.6%)	465(55.4%)	
More than 30min/day	308(45.8%)	44(48.3%)	50(43.1%)	214(46.0%)	
Less than 30min/day	364(54.2%)	47(51.7%)	66(56.9%)	251(54.0%)	
No	537(43.5%)	103(52.3%)	80(40.4%)	354(42.2%)	
**Cancer type**					0.941
DCIS	180(14.6%)	34(17.3%)	27(13.6%)	119(14.2%)	
NST	814(66.0%)	126(64.0%)	133(67.2%)	555(66.2%)	
Lobular	155(12.6%)	25(12.7%)	25(12.6%)	105(12.5%)	
Other	82(6.6%)	12(6.1%)	13(6.6%)	57(6.8%)	
**Cancer subtype**					0.171
Luminal	841(85.2%)	131(88.5%)	143(88.5%)	567(83.6%)	
TNBC	69(7.0%)	7(4.7%)	11(6.8%)	51(7.5%)	
Her2 positive	77(7.8%)	10(6.8%)	7(4.3%)	60(8.8%)	
**Tumoral grade**					0.110
1	250(20.3%)	45(22.8%)	49(24.7%)	156(18.6%)	
2	576(46.7%)	88(44.7%)	88(44.4%)	400(47.7%)	
3	187(15.2%)	21(10.7%)	27(13.6%)	139(16.6%)	
**Treatment**					
**Surgery**	1234(100.0%)	197(100.0%)	198(100.0%)	839(100.0%)	0.741
Mastectomy	283(23.0%)	47(23.9%)	43(21.8%)	193(23.0%)	
Lumpectomy	892(72.4%)	144(73.1%)	146(74.1%)	602(71.8%)	
IBR	57(4.6%)	6(3.0%)	8(4.1%)	43(5.1%)	
**Radiotherapy**					0.269
Yes	991(80.4%)	164(83.2%)	164(82.8%)	663(79.1%)	
No	242(19.6%)	33(16.8%)	34(17.2%)	175(20.9%)	
**Chemotherapy**					0.247
Yes	315(25.5%)	45(22.8%)	44(22.2%)	226(27.0%)	
No	918(74.5%)	152(77.2%)	154(77.8%)	612(73.0%)	
**Hormone therapy**					0.688
Yes	789(64.0%)	121(61.4%)	126(63.6%)	542(64.7%)	
No	444(36.0%)	76(38.6%)	72(36.4%)	296(35.3%)	
**Breast reconstruction**					0.225
Yes	69(5.6%)	16(8.1%)	9(4.5%)	44(5.3%)	
No	1164(94.4%)	181(91.9%)	189(95.5%)	794(94.7%)	

Missing data: Drugs, *n* = 7 (0.6%); Physical activity, *n* = 25 (2%); Cancer Type, *n* = 3 (0.2%); Cancer subtype, *n* = 67 (6%); Tumoral grade, *n* = 41 (3.9%); Surgery, *n* = 2 (0.2%); Radiotherapy, *n* = 1 (0.08%); Chemotherapy, *n* = 1 (0.08%); Hormone therapy, *n* = 1 (0.08%); Breast reconstruction, *n* = 1 (0.08%). Abbreviations: BC, breast cancer; BMI, body mass index; DCIS, ductal carcinoma in situ; NST, no special type; TNBC, triple negative breast cancer; IBR, immediate breast reconstruction. The "*n*" denotes the number of patients. In the case of categorical variables, percentages are expressed between brackets. In the case of continuous variables, the mean value is reported, with standard deviation (SD) between brackets. In the case of non-normal continuous variables, the median value is reported, with interquartile range between brackets (IQR).

Table S2. Characteristics of patients who currently smoke according to smoking assessment by health care professionals at the time of BC diagnosis.

	Patients Who Currently Smoke	Discussed	Not Discussed	*p*-Value
n	197(100%)	78(39.6%)	119(60.4%)	
**Age** (years)	52.7(10.4)	51.6(10.5)	53.5(10.4)	0.226
**BMI** (kg/m2)	23.3(4.2)	23.0(3.8)	23.6(4.4)	0.362
Underweight				0.050
Yes	15(7.6%)	10(12.8%)	5(4.2%)	
No	182(92.4%)	68(87.2%)	114(95.8%)	
**Smoking patterns**				
Age at onset	18.0 [17.0, 20.0]	19.0 [16.0, 22.0]	18.0 [17.0, 20.0]	0.849
Duration(years)	35.6 (12.7%)	33.5 (13.0%)	36.8 (12.3%)	0.083
Amount(cig/day)	12.8 (8.0)	13.8 (8.3)	12.1 (7.8)	0.129
**Cancer type**				0.094
DCIS	34(17.3%)	7(9%)	27(22.7%)	
NST	126(64.0%)	56(71.8%)	70(58.8%)	
Lobular	25(12.7%)	10(12.8%)	15(12.6%)	
Other	12(6.1%)	5(6.4%)	7(5.9%)	
**Cancer subtype**				0.931
Luminal	131(88.5%)	60(89.6%)	71(87.7%)	
TNBC	7(4.7%)	3(4.5%)	4(4.9%)	
Her2 positive	10(6.8%)	4(6.0%)	6(7.4%)	

Missing data: Cancer subtype, *n* = 15 (9.2%). Abbreviations: BC, breast cancer; BMI, body mass index; DCIS, ductal carcinoma in situ; NST, no special type; TNBC, triple negative breast cancer. The "*n*" denotes the number of patients. In the case of categorical variables, percentages are expressed between brackets. In the case of continuous variables, the mean value is reported, with standard deviation (SD) between brackets. In the case of non-normal continuous variables, the median value is reported, with interquartile range between brackets (IQR).

Table S3. Characteristics of patients who currently smoke at BC diagnosis according to smoking cessation status.

	Patients Who Currently Smoke at Diagnosis	Patients Who Had Not Quit	Patients Who Had Quit	*p*-Value
n	197(100%)	132(67%)	65(33%)	
**Age**	52.7 (10.4)	53.2 (11.2)	51.8 (8.7)	0.360
**BMI at Diagnosis** (kg/m2)	23.3 (4.2)	23.3 (4.6)	23.3 (3.4)	0.973
Normal	126 (64.0%)	79 (59.8%)	47 (72.3%)	
Obese	14 (7.1%)	10 (7.6%)	4 (6.2%)	
Overweight	42 (21.3%)	30 (22.7%)	12 (18.5%)	
Underweight	15 (7.6%)	13 (9.8%)	2 (3.1%)	
**BMI at Inclusion** (kg/m2)	24.5 (4.7)	24.5 (5.2)	24.6 (3.8)	0.921
Normal	106(53.8%)	70(53.0%)	36(55.4%)	
Obese	23(11.7%)	17(12.9%)	6(9.2%)	
Overweight	58(29.4%)	39(29.5%)	19(29.2%)	
Underweight	9(4.6%)	6(4.5%)	3(4.6%)	
**Weight difference** (kgs)	+ 2.7 (6.0)	+ 2.1 (5.9)	+ 3.9 (6.0)	0.106
**Smoking patterns**				
Age at onset	18.0 [17.0, 20.0]	18.0 [17.0, 20.0]	18.0 [16.0, 21.0]	0.773
Duration	35.6 (12.7)	36.3 (12.8)	34.3 (12.4)	0.308
Amount (cig/day)	12.8 (8.0)	13.1 (8.2)	12.1 (7.8)	0.396
**Alcohol consumption**				0.585
Yes	78(39.6%)	50(37.9%)	28(43.1%)	
Amount (glass/week)	6.2 (5.8)	6.9 (5.3)	4.9 (5.8)	0.006
No	119(60.4%)	82(62.1%)	37(56.9%)	
**Drugs** (Cannabis or other)				
Current	4(2.1%)	3(2.3%)	1(1.6%)	0.892
Ever	5(2.6%)	3(2.3%)	2(3.1%)	
Never	186(95.4%)	125 (95.4%)	61(95.3%)	
**Physical activity**				0.189
Yes	91 (46.2%)	63 (49.2%)	28 (43.1%)	
More than 30min/day	44 (48.3%)	27 (42.8%)	17 (60.7%)	
Less than 30min/day	47 (51.7%)	36 (57.1%)	11 (39.3%	
No	103(52.3%)	67(51.5%)	36(55.4%)	
**Cancer type**				0.531
DCIS	34(17.3%)	22(16.7%)	12(18.5%)	
NST	126(64.0%)	82(62.1%)	44(67.7%)	
Lobular	25(12.7%)	20(15.2%)	5(7.7%)	
Other	12(6.1%)	8(6.1%)	4(6.2%)	
**Cancer Subtype**				0.407
Luminal	131(88.5%)	92 (90.2%)	39(84.8%)	
TNBC	7(4.7%)	5(4.9%)	2(4.3%)	
HER2-positive	10(6.8%)	5(4.9%)	5(10.9%)	

Missing data: BMI at Inclusion, $n = 1$ (0.5%); Drugs, $n = 2$ (1%); Physical activity, $n = 3$ (1.5%); Cancer Subtype, $n = 15$ (9.2%). Abbreviations: BC, breast cancer; BMI, body mass index; DCIS, ductal carcinoma in situ; NST, no special type; TNBC, triple negative breast cancer. The "n" denotes the number of patients. In the case of categorical variables, percentages are expressed between brackets. In the case of continuous variables, the mean value is reported, with standard deviation (SD) between brackets. In the case of non-normal continuous variables, the median value is reported, with interquartile range between brackets (IQR).
